# The impact of simulation-based mastery learning, booster session timing and clinical exposure on confidence in intercostal drain insertion: a survey of internal medicine trainees in Scotland

**DOI:** 10.1186/s12909-022-03654-7

**Published:** 2022-08-16

**Authors:** Joanne Kerins, Elisabeth McCully, Suzanne Anderson Stirling, Samantha Eve Smith, James Tiernan, Victoria Ruth Tallentire

**Affiliations:** 1 Scottish Centre for Simulation and Clinical Human Factors, Forth Valley Royal Hospital, Larbert, FK5 4WR UK; 2 Queen Elizabeth University Hospital, NHS Greater Glasgow and Clyde, Glasgow, UK; 3 Medical Education, NHS Lanarkshire, Bothwell, UK; 4 NHS Education for Scotland, Glasgow, UK; 5 NHS Lothian, Edinburgh, UK

**Keywords:** Simulation-based mastery learning, Chest drain, Transition from simulation to practice

## Abstract

**Background:**

Intercostal chest drain (ICD) insertion is a skill that medical trainees lack confidence in performing. This study explores the impact of a national programme of Simulation-Based Mastery Learning (SBML) on procedural confidence, including the impact of time intervals between booster sessions and interim clinical experience.

**Methods:**

Internal Medicine Trainees in Scotland were surveyed about confidence and clinical experience with ICD insertion before and immediately after SBML and booster session. Data were matched and analysed using paired sample t-tests. Short interval and long interval groups were compared using Student’s unpaired t-test. The impact of interim clinical experience was assessed using Analysis of Variance.

**Results:**

Mean confidence in ICD insertion rose following SBML, fell between initial and booster session, and increased again following booster session (*P* =  < 0.001). 33 of 74 trainees had successfully inserted an ICD between sessions. Fall in confidence was unaffected by the time interval between training sessions, but was mitigated by interim clinical experience.

**Conclusions:**

SBML boosts trainee confidence in ICD insertion. However, there is evidence of confidence decay, possibly due to a lack of clinical experience between sessions. More research is needed to explore barriers to transfer of skills from simulated to real-world environments.

## Background

Intercostal chest drain (ICD) insertion is a potentially life-saving, complex procedural intervention, usually performed in an acute hospital environment. ICD insertion can have significant adverse outcomes including iatrogenic pneumo/haemothorax, infection, organ puncture and, rarely, death [1]. Previous studies have suggested that doctors in training are becoming less competent and less confident at inserting ICDs [2–6]. If these skills are taught but unused, they will decay over time [7]. ‘Skill decay’ is defined as the degradation or loss of trained or acquired skills, after a period of non-use [7]; with greater time intervals between use associated with greater loss of skill [8]. Given ICD insertion comes with significant risk of complications, understanding how best to train doctors in becoming competent and confident in this skill is key for medical educators.

The relationship between confidence and competence is complex, but both are important in the acquisition of new skills [9]. Stewart et al*.* suggest that competence is defined by what one is able to do (incorporating an element of external validation of ability), while confidence can be defined as a belief in one’s ability to complete a task (incorporating one’s level of anxiety) [10]. There is also the related motivational construct of self-efficacy, the judgements that trainees make about their capabilities, [11] which is sometimes referred to as task-specific self-confidence [12]. Ideally, confidence and competence should align such that the more competent one is, the more confidence one has. Although many have argued that confidence or self-efficacy can be used as a proxy for performance, [11, 13–15] previous studies have shown that confidence and competence correlate poorly [16–19]. Despite this, confidence remains important. Confidence impacts willingness to undertake procedures [2] with a lack of confidence leading to avoidance of practising technical skills in the workplace [20]. There is also evidence that self-efficacy positively influences the successful transfer of training of skills to the workplace [21]. It is therefore crucial that doctors in training develop sufficient confidence in essential procedures in order to seek opportunities to practice their skills, which should ultimately lead to improved competence and better patient outcomes.

Simulation-based mastery learning (SBML) is a robust, evidence-based teaching methodology designed to improve the acquisition of procedural skills, with the aim of supporting all learners to achieve an agreed standard [22, 23]. The premise of SBML is that all learners can achieve the same level of competence, but that different time periods or repeated application of deliberate practice may be required [22]. It is well evidenced that SBML has significant advantages for acquiring competence in new skills, as it can ensure a standardisation of experience, provide a learning environment with no risk to patients, provide safety for the novice learner, and bridge the gap between clinically available opportunities and participants’ learning needs [22]. One-off simulation sessions have been shown to improve competence, confidence, and reduce ICD insertion complication rates [24–26].

However, after such simulation sessions, skill decay inevitably occurs. A previous meta-analysis of skill decay literature showed substantial loss of skill after 365 days of non-use [7]. More recent studies have suggested that skill decay can begin much earlier than previously thought [27–30]. SBML booster sessions can be utilised to mitigate skill decay, [31–33] but debate remains as to the best timing of such sessions. In addition, there is a lack of research exploring the decay of confidence in procedural skills following SBML, which could influence trainees’ willingness to undertake procedures in the workplace [2]. Confidence decay is expected after non-use, but some trainees will have the opportunity to transfer procedural skills to the workplace. The impact of such clinical exposure between SBML sessions on confidence is underexplored and could influence training needs. A better understanding of confidence decay following SBML sessions could inform the need for and timing of booster sessions. Given the evidence of substantial skill decay after 12 months, we aimed to explore whether confidence decays after a similar interval, and to determine the impact of clinical exposure in the intervening period.

### Purpose of the study

We aimed to explore the relationship between SBML and confidence in ICD insertion, specifically:

1. What impact (if any) does SBML have on ICD insertion confidence?
2. What impact (if any) does a booster session have on ICD insertion confidence?
3. Is there a difference in level of confidence decay in ICD insertion between short interval to booster (< 12 months) versus long interval to booster (12 months or more) groups?
4. What is the impact (if any) of practising in the workplace on ICD insertion confidence decay? Specifically:

- a. Does decay of confidence differ when participants have clinical experience in the workplace have a long or short interval to booster, versus if they have no clinical experience?
- b. Is there a relationship between number of successful attempts in the workplace and decay in confidence?

## Methods

### Context

In the UK, Internal Medicine Training (IMT) is a three-year national programme for doctors who have at least two years of post-graduation clinical experience. Those wishing to apply for higher level training in a medical speciality must complete IMT or prove equivalent competences via an alternative route. IMT in Scotland incorporates a national simulation strategy, which includes a three-day boot camp during the first year of IMT (IMT1), with mixed groups from all four Scottish regions (West, North, East and South-East) hosted at the Scottish Centre for Simulation and Clinical Human Factors. In the second year of the training programme (IMT2) trainees attend a skills day at either the Royal College of Physicians and Surgeons of Glasgow (West and South-East trainees), or Aberdeen Royal Infirmary (North and East trainees), to revisit some of the skills covered at boot camp in IMT1.

The SBML pathway in this context involves study of pre-learning materials and peer assisted deliberate practice, followed by a checklist-based summative assessment to ensure the passing standard is reached [23]. Practical procedures covered using SBML include ICD insertion, lumbar puncture, ascitic drain insertion and central venous access [23]. ICD insertion (guidewire technique) is formally assessed at both IMT1 boot camp and IMT2 skills day, using an identical checklist-based process encompassed within a two hour session. The learning materials used during these training events were created by NHS Lothian and are available on the Medical Education Directorate website [34].

### Data collection

Participants completed evaluation questionnaires before and immediately after each of the two training events (IMT1 boot camp and IMT2 skills day), and data was tracked for each participant across both courses. At the time of completing each survey, participants consented to their anonymised data being used for course improvements and research. Pre and post event, participants were asked to consider their clinical practice and answer the following question:

*‘How confident are you that you could safely and successfully perform the following procedures under direct supervision (with a consultant at the end of the bed)?’*

They were asked to self-rate confidence on a Likert scale from 1 to 7 where 1 = not at all confident and 7 = completely confident. Participants were also asked how many times they had attempted each procedure (successfully and unsuccessfully) in clinical practice (including all supervised or independent attempts), between training events. The time interval between courses for each participant was calculated.

### Data analysis

The Scottish national IMT simulation strategy provides one simulation training course per trainee for each training year. However, IMTs can attend any course within that training year, and are currently randomly assigned, leading to variability in time interval between participants attending these courses. As mentioned, previous meta-analysis of skill decay literature has shown substantial loss of skill after 365 days of non-use [7]. We aimed to determine if those who had a less than 12 month interval between training events, had a significant advantage over those with a greater than 12 month interval. This would either support maintaining one intervention per training year, or provide evidence for a need to reform our training programme to incorporate more frequent intervention. Electronic evaluation survey data was collected from participants pre and post both events between August 2019 and May 2021. The relevant data was anonymised by use of participant codes, matched to track self-reported confidence changes across the two events.

Confidence change was calculated by subtracting the post- IMT1 boot camp Likert score for confidence from the pre- IMT2 skills day (booster session) Likert score for confidence. A negative value for confidence change indicated confidence decay. The data was analysed using SPSS (version 14) and Excel (version 2205). The following statistical tests were performed:

- Aim 1: Differences in confidence before and after the initial SBML session were compared using a paired samples t-test
- Aim 2: Differences in confidence before and after the booster session were compared using a paired samples t-test
- Aim 3: We examined change in confidence between the post-initial session score and the pre-booster session score. The confidence decay between short interval (< 12 months) and long interval (> = 12 month) groups were compared using Student’s unpaired t-test
- Aim 4a: Confidence decay between short and long interval groups, split by clinical experience of performing ICD insertion in the workplace versus no experience were compared using Analysis of Variance.
- Aim 4b: Confidence decay between groups who were successful in ICD insertion in the workplace between the initial course and the booster were compared using Analysis of Variance.
- Differences were considered statistically significant if *p* < 0.01 (5% significance level with a Bonferroni correction for five comparisons).

## Results

One hundred and five trainees attended the IMT1 boot camp between August 2019 and January 2020, and 100 trainees subsequently attended the IMT2 skills day between September 2020 and May 2021. Seventy-four participants had complete data sets for the data extracted with regard to ICD insertion, allowing tracking of matched data from IMT1 boot camp to IMT2 skills day. Twenty-six participants were excluded due to incomplete survey responses. This was the only exclusion criterion. Demographic data for the 74 included participants is included below in Table 1.

**Table 1:** Demographic data for participants

Gender	**Male**		**Female**	
Trainees	34		40	
Age range	**24–29**	**30–35**		**36 and over**
Trainees	66	7		1
Region	**East**	**North**	**South East**	**West**
Trainees	8	10	18	38

### Aims 1 and 2: Impact of SBML and the booster session on confidence

**Fig. 1:**
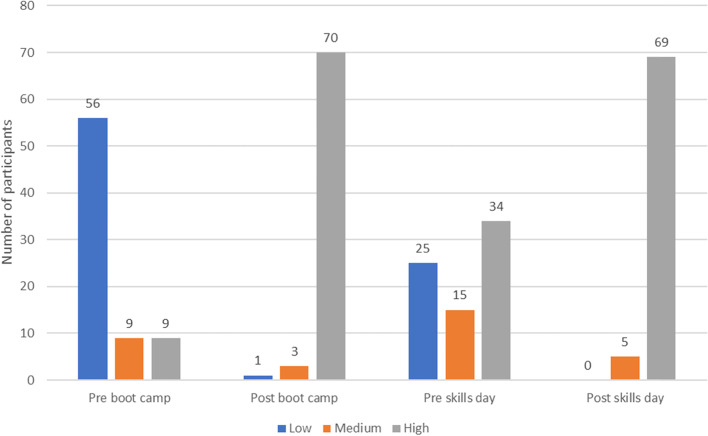
Number of participants who reported low, medium or high confidence at different stages of their training

Prior to SBML, the mean score for self-reported confidence in ICD insertion was low (mean of 2.6 out of 7). There was a statistically significant increase (*p* < 0.001) in mean confidence following the boot camp initial SBML session (from mean confidence 2.6 to 5.9 out of 7). The mean confidence level then decreased following the interval between boot camp (initial) and skills day (booster). The mean self-reported confidence from subsequent skills day data was 4.2 out of 7, rising to 5.9 out of 7 following the session, and this rise was again statistically significant (*p* < 0.001). This is further exemplified in Fig. 1 which shows a breakdown of self-reported confidence at each stage with responses grouped into low confidence (Likert response 1–3), medium confidence [4] and high confidence [5–7].

### Aim 3: Impact of booster session timing on confidence decay

**Fig. 2:**
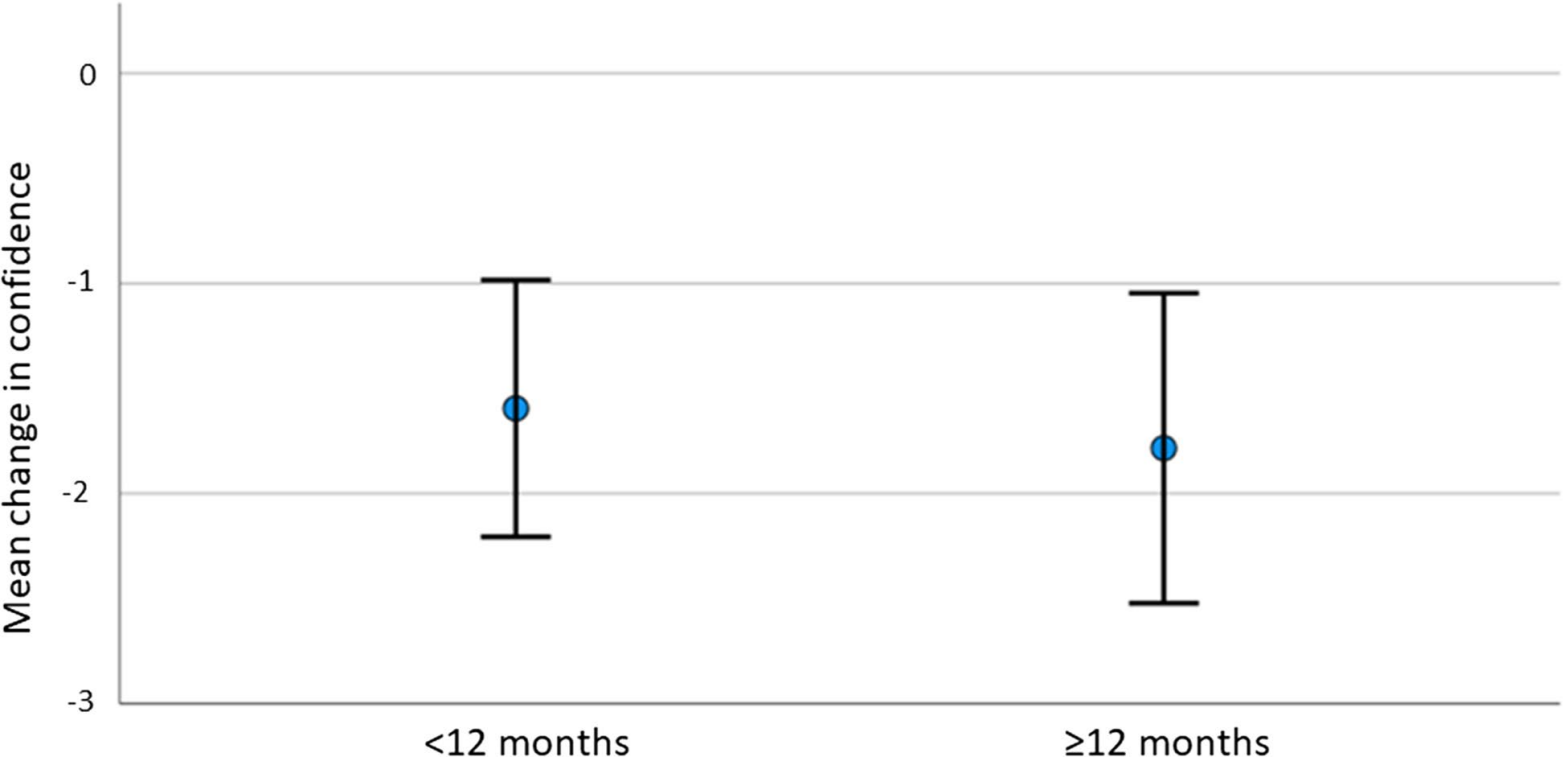
Confidence decay in groups with a short interval (< 12 months) versus long interval (> = 12 months) between initial session and booster session

There was no statistically significant difference (*p* = 0.69) in confidence decay between groups with a short time interval between the initial SMBL and booster session (confidence decay -1.59) versus groups with a long time interval of >  = 12 months (confidence decay -1.78), as depicted in Fig. 2.

### Aim 4a: Confidence decay in short and long interval groups grouped according to level of clinical experience

**Fig. 3:**
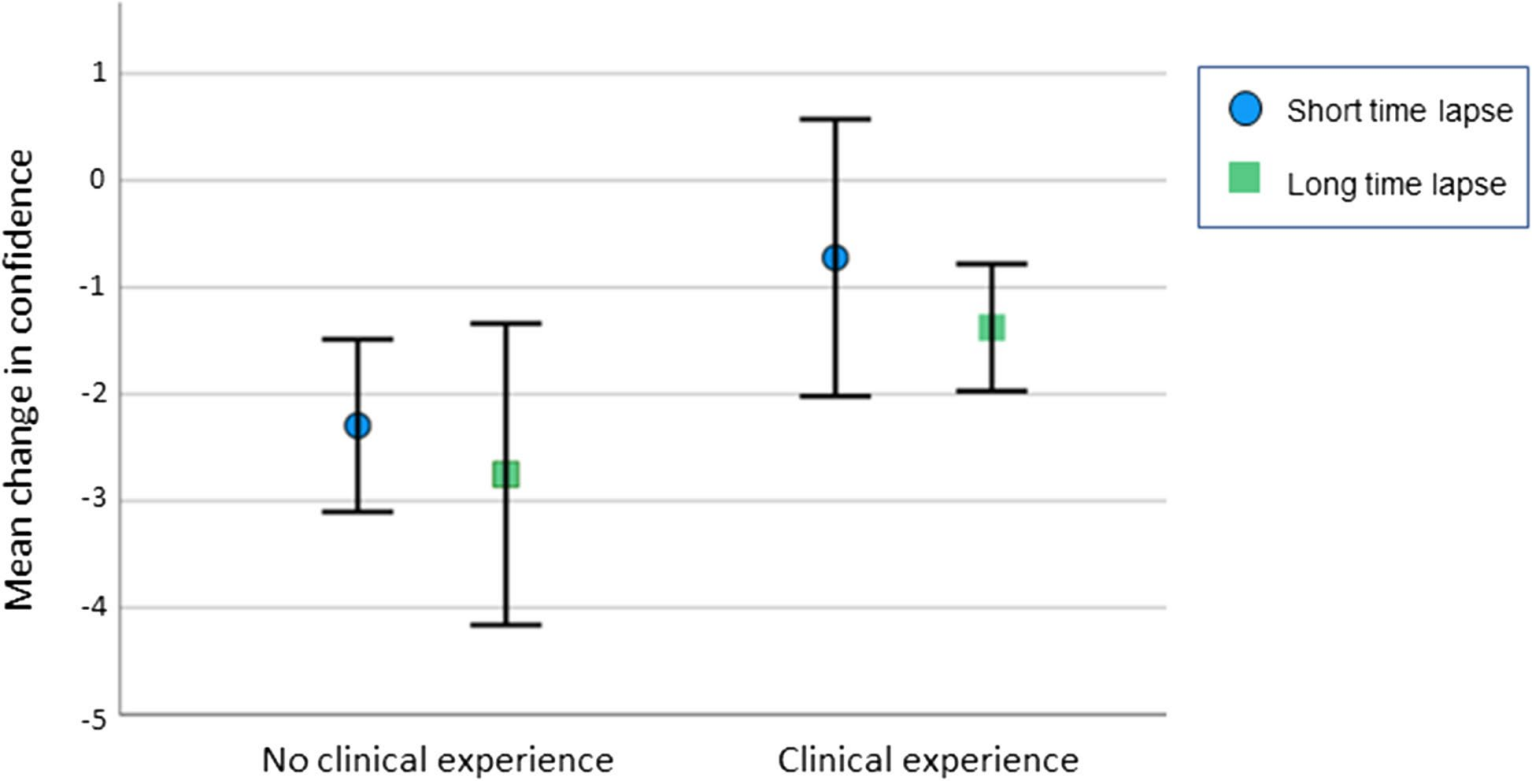
Confidence decay according to clinical experience and time interval between initial session and booster session

Figure 3 shows the differences between the four groups. The trend is towards greater confidence decay in the groups with no clinical experience, however within this small study the results are not statistically significant (*p* = 0.023).

### Aim 4b: Relationship between successful clinical attempts between the initial and booster sessions and confidence decay

Fewer than half (44%) of the participants successfully inserted an ICD in clinical practice between the IMT1 boot camp and the IMT2 skills day. The breakdown of number of successful attempts of participants is displayed in Table 2.

**Table 2:** Number of successful ICD attempts between initial and booster session

Number of successful ICD insertion attempts	Number of participants
None	41
1 -3	21
4—6	6
7—10	5
> 10	1

**Fig. 4:**
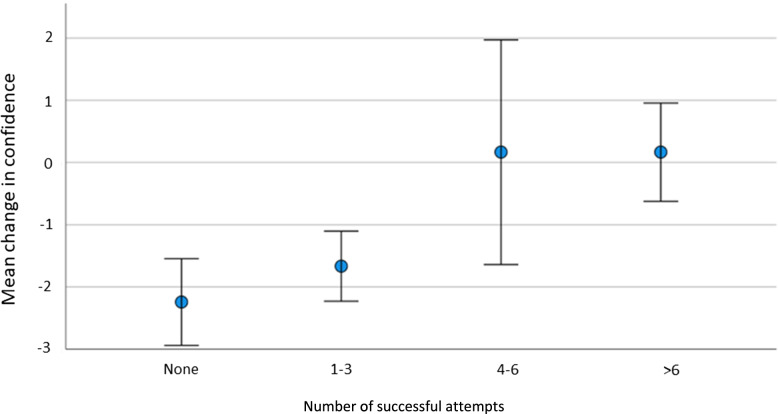
Difference in confidence levels of groups with differing numbers of successful attempts at ICD insertion between the initial and booster training sessions

Confidence decay appeared to be ameliorated by successful ICD insertion attempts in clinical practice between the initial and booster sessions (as shown in Fig. 4), and there was a statistically significant difference within the data (*p* = 0.003). The dataset was not sufficiently large to assess the number of successful insertions needed to reduce confidence decay, but does show that there is a definite association between higher numbers of successful attempts and reduction in confidence decay.

Only eight participants recorded unsuccessful attempts. Of these, six recorded 1–3 unsuccessful attempts and two recorded 4–6 unsuccessful attempts. Six of those with unsuccessful attempts also recorded successful attempts.Interestingly, the two participants with unsuccessful attempts but no successful attempts did not report a confidence decay.This dataset is too small to draw any conclusions about the effect of unsuccessful attempts on confidence, but it would be an interesting question for a future study.

## Discussion

This study explores the impact of a national structured SBML programme on trainee confidence in ICD insertion, and how this is impacted by the time interval between training sessions and intervening clinical practice. This study shows that SBML for ICD insertion improves trainee reported confidence. Mean confidence fell between sessions but was improved with a booster session, with no significant difference in confidence decay if the booster session was less than or more than 12 months after the initial session. Although confidence fell even when trainees had clinical experience in the interim, there was some mitigation of the confidence decay with clinical experience.

These findings are congruent with the extensive evidence basis for the use of SBML, which has been shown to improve participant confidence [15, 22, 24, 35–38]. This longitudinal study adds to the SBML literature by exploring change in confidence over time and shows evidence of confidence decay. The ultimate aim of training interventions is to transfer skills to the workplace and trainees’ confidence in their own abilities to do so is a key factor in the transfer of skills being successful [21]. Although trainees may be deemed competent to perform procedures on a manikin in a skills lab, there are numerous factors in the work environment that can influence skill transfer [39]. If trainees have persistent low self-efficacy for skills they feel they should be confident and competent to perform in their role, then this could lead to discomfort or anxiety [40]. Previous studies have found that internal medicine residents were uncomfortable with bedside medical procedures [40, 41] and, in particular, uncomfortable with the skill of ICD insertion [40]. Training programmes should aim to improve and maintain confidence in essential skills in order to ensure opportunities are sought and a potential cycle of avoidance and further reduced confidence prevented. SBML in this context can boost confidence but how best to maintain this for trainees requires further consideration.

Trainees must have access to clinical opportunities to perform in order to transfer skills to the workplace which, as exemplified in this study, is challenging, particularly for the skill of ICD insertion [39]. This study found significant mitigation of confidence decay for trainees with higher numbers of successful attempts in clinical practice during the intervening period, in keeping with other studies finding that clinical practice increases confidence in ICD insertion [2, 6, 42, 43]. However, less than half of the trainees had successfully inserted an ICD during the interim time interval. It is possible that the COVID-19 pandemic, during which ICD insertion for pneumothorax was classified as an ‘aerosol generating procedure’, requiring enhanced personal protective equipment, may have contributed to the lack of opportunities over the study period. It is possible that the majority of our participants had not reached a “threshold” number of drain insertions which would maintain their confidence [44]. Studies exploring competence have suggested that the number of ICD insertions needed to gain and subsequently maintain competence may be between five and ten per year [3, 44]. The trend within this dataset would suggest that approximately 4–6 may be the threshold to ameliorate confidence decay between sessions. Given the low number of ICD attempts between sessions for our participants, the clinical implications of achieving this number, and therefore maintaining the confidence of each trainee, presents major challenges.

## Implications for practice

Unlike the findings in other published literature, [45] time interval between training sessions within the parameters of this study had no significant impact on the degree of confidence loss. Based on these findings, there appears to be no benefit in shortening the time interval between training days to less than one year. This study suggests that, for those considering implementation of a similar SBML training programme, one training session per training year will boost confidence. However, there may be an argument to consider reducing the interval between sessions for trainees who have not had the opportunity to perform ICD insertion in clinical practice. The results of this study add to the evidence for SBML, and support the ongoing use of the IMT2 skills day as a booster session to support confidence in ICD insertion. It raises the question of whether booster session trainee allocations should be based on clinical experience.

## Strengths and limitations

This national study tracked participants across two training sessions, using longitudinal data over two years, providing a strong evidence base for reviewing the training programme and altering practice. There are limitations in measuring self-reported confidence as we know this may not correlate well with competence, for example trainees may be over-confident but not competent or a lack of confidence may not reflect lack of competence. However, given all trainees achieved competence during the assessed session, individual self-reported confidence was deemed an important indicator of their self-efficacy in transferring skills to the workplace. It must also be recognised that there are other factors which could influence confidence in performing ICD insertion, such as the precise timing of clinical experience between sessions and supervisor support during such experience, that have not been addressed in this study [39].

## Future work

Future work could explore the barriers to transfer of procedural skills to the workplace, including opportunities to perform from trainee, supervisor and organisational perspectives. Recent work has identified factors such as opportunities to perform the procedure clinically and support from supervisors as crucial to facilitating successful transfer [39, 46]. While our results indicate that intervals of 7–20 months between sessions have little impact on trainee confidence, further work is needed to evaluate if shorter intervals of a few months between SBML training interventions maintain confidence or indeed promote transfer to clinical practice. With a larger powered study, the time intervals between sessions could be further subdivided to explore shorter booster session timing impact on confidence. It would also be helpful to assess if findings are similar in relation to other important but infrequently used procedural skills, other than ICD insertion.

## Conclusions

This study reinforces the need for SBML and booster sessions for ICD insertion, particularly given the challenges of obtaining clinical exposure for internal medicine trainees. The finding of confidence decay being mitigated by successful attempts in clinical practice between training sessions should be of interest to medical educators involved in the training of important but infrequently used skills. Further research into opportunities to perform in the workplace is needed to establish how best to support trainees transferring this skill into clinical practice in order to maintain confidence.

## Data Availability

The datasets analysed during the current study are not publicly available as they are part of a larger data set which may be subject to future publication, and contains information that could compromise individual privacy. Data are available from the corresponding author on reasonable request.

## Abbreviations

ICD: Intercostal Drain
IMT: Internal Medicine Training
SBML: Simulation-Based Mastery Learning
UK: United Kingdom
